# Aging-associated alterations in epidermal function and their clinical significance

**DOI:** 10.18632/aging.102946

**Published:** 2020-03-27

**Authors:** Zhen Wang, Mao-Qiang Man, Tienan Li, Peter M. Elias, Theodora M. Mauro

**Affiliations:** 1 Shenyang No.7 People’s Hospital, Shenyang 110003, Liaoning, China; 2 Dermatology Hospital, Southern Medical University, Guangdong 510091, China; 3 Department of Dermatology, University of California San Francisco and Veterans Affairs Medical Center, San Francisco, CA 94121, USA

**Keywords:** epidermal permeability barrier, aging, emollient, pH, hydration

## Abstract

Chronologically-aged skin displays multiple functional changes in both the dermis and the epidermis. It appears that epidermal dysfunction, compromised permeability homeostasis, reduced stratum corneum hydration and elevated skin surface pH predispose to the development of aging-associated cutaneous and extracutaneous disorders. Improvements in epidermal function have been shown to be an effective alternative therapy in the prevention and treatment of some aging-associated cutaneous disorders, including eczematous dermatitis, pruritus, and xerosis. Recent studies demonstrated that epidermal dysfunction leads to the development of chronic, low-grade systemic inflammation, termed ‘inflammaging,’ which is linked to the development of aging-associated systemic disorders. Thus, correction of epidermal dysfunction could comprise a novel strategy in the prevention and treatment of aging-associated systemic disorders as well. In this review, we summarize aging-associated alterations in epidermal function, their underlying mechanisms, and their clinical significance. Regimens to improve epidermal function in the elderly are also discussed.

## INTRODUCTION

With advances in medical biology and healthcare technology over recent decades, human lifespans are increasing worldwide, with lifespan expectations of up to 100 years in developed countries by 2025 [1], resulting in a proportionate increase in the aged population. As early as 50 years of age, the frequency of aging-associated cutaneous disorders increases, in parallel with epidermal dysfunction, including compromised permeability homeostasis and reductions in levels of stratum corneum hydration, as well as elevations in skin surface pH, the most prominent features associated with chronic aging. Studies have shown that epidermal dysfunction, in turn, predisposes to the development of a variety of cutaneous abnormalities, including atopic dermatitis, contact dermatitis, pruritus and xerosis, and possibly aging-associated systemic disorders [2–5]. In this review, we discuss aging-associated alterations in epidermal function and their link to cutaneous disorders.

## Aging-associated alterations in epidermal function

### Compromised epidermal permeability barrier homeostasis

Aging-associated changes in baseline transepidermal water loss (TEWL) rates, an indicator of epidermal permeability barrier, vary greatly with gender, body sites and pigment types. While some studies have shown that baseline TEWL rates on several body sites are lower in the aged than in young skin [6–12], other study demonstrated that TEWL rates on the décolleté region correlated positively with age, but TEWL rates on the neck, forearm and hand were comparable between young and aged women [13]. Moreover, TEWL rates are higher in aged females than in aged males [10]. Yet, in both aged humans and mice, following acute disruption of permeability barrier function, permeability barrier recovery is significantly delayed in comparison to younger age groups [7, 14]. In addition, stratum corneum integrity also decreases in both aged humans and mice [7]. Taken together, aged epidermis displays defects in permeability barrier homeostasis.

Several alterations in the aged skin contribute to a defective permeability barrier function. The epidermal permeability barrier resides in the stratum corneum, the outermost layer of the epidermis. According to the ‘brick and mortar’ model, this permeability barrier is largely determined by quality and quantity of differentiation-related proteins and extracellular lipids in the stratum corneum. Previous studies demonstrated that levels of epidermal growth factor reduced in parallel with a decline in keratinocyte proliferation in the aged epidermis, while keratinocyte apoptosis increased, leading to reductions in the thickness of both the epidermis and the stratum corneum [15–17]. Because high calcium concentration inhibits human keratinocyte proliferation [18], thinning epidermis could also be attributed to an increased calcium gradient in the basal and spinous layers [19], where the keratinocyte proliferation is most active in the epidermis. Moreover, levels of structural proteins for the epidermal permeability barrier, including filaggrin, loricrin and other late cornified envelope proteins, markedly decline in aged skin in comparison to young skin [20–22], perhaps due to reductions in calcium content in the stratum granulosum, leading to defective ‘bricks’ [21]. Deficiencies in these proteins can result in a defective permeability barrier [3].

In addition to such defective ‘bricks,’ reductions in production of the lipid-enriched ‘mortar,’ i.e., the epidermal lipids, are also evident in the aged epidermis. Because formation of a competent epidermal permeability barrier requires an approximately equal molar ratio of cholesterol, free fatty acids and ceramides [23, 24], which are synthesized by epidermal keratinocytes [25, 26], deficiencies in any of these lipids can result in a defective epidermal permeability barrier [25]. Prior studies have shown that the aged stratum corneum displays a >30% reduction in total lipid content in comparison to young stratum corneum [7], due to reduced epidermal lipid synthesis, particularly in cholesterol synthesis, both under basal conditions and after barrier disruption [14]. Notably, aging-associated reduction in ceramide 2 was only observed in females, not in males, although ceramide levels did not differ significantly between aged males and females [27]. In support of evidence that reduced lipid levels contribute to aging-associated dysfunction in epidermal permeability barrier, topical applications of stratum corneum physiologic lipid mixtures can improve epidermal permeability barrier function in aged humans and mice [28]. Thus, these reductions in lipid production and differentiation marker-related protein levels could be causing the compromised epidermal permeability barrier homeostasis in aged skin. The epidermal permeability barrier is also largely made up of extracellular multilamellar bilayers, whose formation requires enzymatic processing of lipid precursors within the extracellular spaces of the stratum corneum [29–31]. The optimal pH for these enzyme activities is ≈5.0 [30, 31]. Yet, aged skin manifests an elevation in skin surface pH in comparison to young skin [32–34]. While topical applications of buffers at neutral pH delay barrier recovery [35], acidification of stratum corneum accelerates barrier recovery in both young and aged murine skin [34, 36, 37]. Hence, the elevated stratum corneum pH of aged skin likely contributes to the delay in permeability barrier recovery.

Chronological aging is accompanied by an increase in glucocorticoid secretion and cortisol content in the skin [15, 38], which can cause epidermal dysfunction. Previous studies have shown that either systemic or topical applications of glucocorticoids compromise epidermal function, including permeability barrier homeostasis and epidermal proliferation [39, 40]. Moreover, glucocorticoid action requires the peripheral conversion of cortisone to cortisol by 11β hydroxysteroid dehydrogenase 1 [41]. In comparison to young skin, aged skin exhibits higher levels and activity of 11β hydroxysteroid dehydrogenase 1 [42], and this epidermal 11β hydroxysteroid dehydrogenase 1 activity correlates negatively with epidermal permeability barrier function [43]. Conversely, inhibition of 11β hydroxysteroid dehydrogenase 1 not only corrects glucocorticoid-induced epidermal functional abnormalities, but also improves aging-associated structural and functional alterations [44, 45]. Thus, the aging-associated increase in epidermal 11β hydroxysteroid dehydrogenase 1 and cortisol content can contribute to defective permeability barrier function in aged skin.

**Figure 1:**
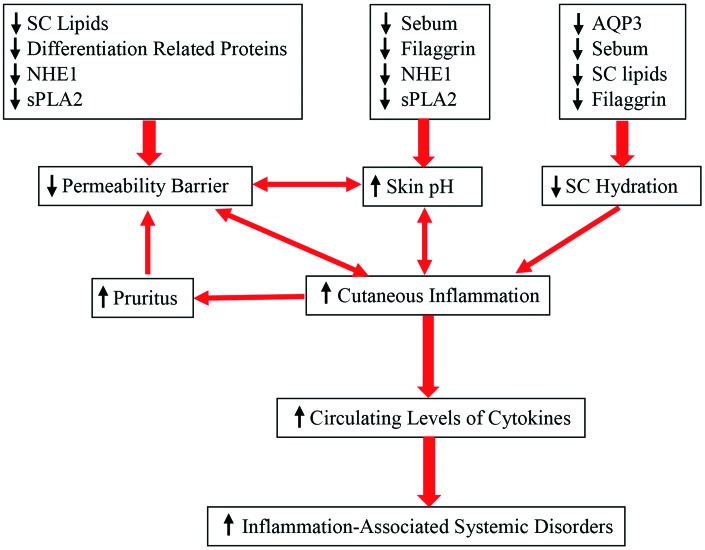
**Aging-associated changes in epidermal function and their clinical significance.**

Additionally, other aging-associated changes in the skin can also contribute to altered epidermal function. For example, the aged epidermis displays over 60% reduction in IL-1 receptor antagonist protein in comparison to young epidermis, and a deficiency in IL-1α receptor type 1 delays barrier recovery [46]. Conversely, either upregulation or administration of IL-1α enhances epidermal permeability barrier function in both aged and fetal skin [47, 48]. Similarly, aged skin also exhibits reduced levels of hyaluronic acid [49]. Previous studies have shown that topical applications of hyaluronic acid stimulate keratinocyte differentiation and lipid production, leading to enhancement of epidermal permeability barrier function in both young and aged skin [50, 51]. Finally, aging-associated reductions in epidermal aquaporin 3 expression have also been observed [52–54], while knockout of epidermal aquaporin 3 delays permeability barrier recovery [55]. Conversely, upregulations of epidermal aquaporin 3 expression improve epidermal permeability barrier function [54, 56]. Collectively, aged epidermis displays multiple alterations in keratinocyte function, including altered signaling pathways of calcium, cytokine and hyaluronic acid, stratum corneum acidification, keratinocyte proliferation, differentiation, lipid production, as well as decreased epidermal aquaporin 3 expression, consequently leading to compromised epidermal permeability barrier function (Figure 1).

### Reduction in stratum corneum hydration

In humans, stratum corneum hydration over a lifetime increases to a peak level at age 40 years, followed by a decline, especially on the face and neck in males [10, 32, 57, 58]. The age-dependent differences in hydration are most prominent at a depth of 10-30 μm (on the forearm) in the stratum corneum [59]. It also appears that age-dependent changes in stratum corneum hydration vary with ethnicity. For example, the skin dryness index on the forearm markedly increases in aged African-American and Caucasian skin, but not in aged Chinese and Mexican skin, in comparison to young people of the same ethnicity [60]. The mechanisms underlying reduced stratum corneum hydration in the aged skin can be ascribed to the reduced content of natural humectants in the skin. Firstly, lipid content decreases in the stratum corneum of aged skin [7, 14, 61, 62]. Among these stratum corneum lipids, ceramides exhibit water-holding properties [63]. Prior studies have demonstrated that either oral or topical administration of ceramides can increase stratum corneum hydration [64, 65]. Secondly, aged epidermis exhibits reduced levels of filaggrin [22] and its metabolites, including trans-urocanic acid and pyrrolidone carboxylic acid, which are natural moisturizers in the skin [66]. Thirdly, both sebum and glycerol contents are reduced in aged versus young skin [32, 67]. Deficiency in either sebum or glycerol decreases stratum corneum hydration [68, 69], while topical applications of glycerol improve stratum corneum hydration [56, 69–71]. Finally, levels of aquaporin 3 decrease in aged versus young epidermis [53–55], leading to reduction in stratum corneum hydration. Aquaporin 3 deficiency-induced reduction in stratum corneum hydration is likely due to decreased glycerol content in the stratum corneum [70, 71]. Accordingly, upregulation of epidermal aquaporin 3 expression or topical glycerol improves stratum corneum hydration in aquaporin 3-deficient mice [71, 72]. Thus, aging-associated reductions in stratum corneum hydration can be attributed, in large part, to a reduced content of natural moisturizers in the epidermis (Figure 1).

### Elevation in skin surface pH

In humans, skin surface pH is generally higher in the first 2 weeks of life, followed by a decline by 5-6 weeks old [73]. Skin surface pH begins to increase at 55 years of age [32, 34]. Marked elevations in skin surface pH occur in aged humans, particularly in those over 70 years old [32, 34, 74–76]. Human skin surface pH varies with gender and body site. For example, skin surface pH on the upper eyelid is 5.13 ± 0.49 (mean ± SD), and 5.75 ± 43 (mean ± SD) on the forearm in subjects aged 66-83 years [76]. Similarly, the skin surface of the abdomen displays a higher pH than that of the upper back [8]. In males [but not females], the highest skin surface pH was found on the forehead and the forearm in subjects over 70 years of age [32]. Moreover, skin surface pH, at least on the forehead, forearm, cheek and hand, is higher in aged females than in aged males [10, 32]. However, skin surface pH is comparable between males and females on both the axillary vault and fossa [76].

In terms of etiology, at least four factors can contribute to the aging-associated elevation in skin surface pH. One is the sebum content which declines in aged skin [13, 32], resulting in reduced triglycerides in the stratum corneum. Degradation of triglycerides yields free fatty acids, which can acidify stratum corneum [77]. Likewise, generation of free fatty acids from phospholipids by secretory phospholipase 2 [sPLA2] can also acidify the stratum corneum [78]. Expression levels of sPLA2 markedly decreased in aged skin [79]. Thus, aging-associated reduction in sebum and sPLA2 levels can contribute, at least in part, to the elevated skin surface pH in aged skin. Sodium-hydrogen exchanger 1 (NHE1) is another contributor to elevated skin surface pH in aged skin. Prior studies demonstrated that NHE1 deficiency increased skin surface pH in mice [80], while aged skin, at least in mice, exhibits significantly lower expression levels of NHE1 in comparison to young skin [79]. Hence, elevated skin surface pH in aged skin can be due to reduction in epidermal NHE1 expression as well. In addition, aged epidermis displays low expression levels of filaggrin [21], which can be degraded to trans-urocanic acid via a filaggrin-histidine-urocanic acid pathway [81]. Urocanic acid content in the stratum corneum correlates positively with skin acidity [82]. Collectively, reductions in sebum content and levels of NHE1, sPLA2 and filaggrin can contribute to aging-associated elevation in skin surface pH (Figure 1).

## Consequences of aging-associated alteration in epidermal function

### Reduced stratum corneum hydration

Reductions in stratum corneum hydration have been implicated in the pathogenesis of senile xerosis and aging-associated pruritus [83, 84]. Previous studies have demonstrated that reductions in SC hydration increase inflammatory infiltration, mast cell density, mast cell degranulation, and histamine content in mouse dermis [85, 86]. Increased cytokines and histamine in the skin can provoke scratching due to pruritus, resulting in disruption of epidermal permeability barrier, consequently leading to a further increase in cutaneous inflammation. Because the epidermal permeability barrier homeostasis in aged skin is compromised [7, 14], it cannot be rapidly normalized, leading to a sustained increase in cutaneous inflammation and exacerbation of preexisting inflammatory conditions, such as atopic dermatitis and eczema. Moreover, the sustained increase in cutaneous inflammation could eventually cause systemic inflammation, possibly leading to the development of inflammaging-associated disorders [5, 87]. Additionally, nocturnal pruritus can cause insomnia, resulting in exacerbation of other disorders, such as cardiovascular and Parkinson diseases [88, 89]. Thus, reduced stratum corneum hydration can lead to the development of both cutaneous and extracutaneous disorders.

### Dysfunction in epidermal permeability barrier

As mentioned above, permeability barrier recovery is delayed in aged skin, although the baseline permeability barrier is comparable to young subjects. Disruption of epidermal permeability barrier alone not only increases expression levels of cutaneous cytokines [90], but also increases inflammatory infiltration in the skin [91–94], leading to the development of cutaneous inflammation and pruritus. Moreover, when the permeability barrier is disrupted by scratching or other forms of insults, the epidermal ‘window’ would keep opening long enough to let harmful substances penetrate the skin, because of delayed barrier recovery in aged skin. Consequently, aged skin becomes vulnerable to the development of atopic dermatitis and contact dermatitis [95, 96]. Again, sustained cutaneous inflammation can provoke systemic inflammation. Finally, a defective permeability barrier favors bacterial colonization in the skin [97, 98]. Thus, aging-associated dysfunction in epidermal permeability barrier can contribute to the development of cutaneous infections, pruritus, dermatitis, and possible systemic inflammation.

### Elevated skin surface pH

Elevated skin pH can impact several aspects of cutaneous function. First, lamellar membrane bilayers in the stratum corneum are the critical structures of the epidermal permeability barrier. Formation of mature membrane bilayers requires processing of lipid precursors by several enzymes, including beta-glucocerebrosidase, acidic sphingomyelinase, and acidic secretory phospholipase A2, with an optimal pH range of 4.5 to 5.2 [30, 31]. Hence, elevated skin pH can abrogate the maturation of membrane bilayers, resulting in a compromised epidermal permeability barrier. Secondly, the antimicrobial properties of the skin are pH-dependent [99]. Certain pathogens, such as *Staphylococcus aureus* and fungi, favor a neutral pH, while an acidic pH decreases survival ability of *Staphylococcus aureus* [100–102]. Thus, the increased skin surface pH can contribute to a high prevalence of cutaneous infections in the elderly. Thirdly, the epidermis is rich in proteases with either optimal basic or acidic pH. For example, stratum corneum cathepsin-like protease, with an optimal acidic pH, degrades corneodesmosin, a component of corneodesmosomes [103]. Elevated stratum corneum pH can decrease the activity of cathepsin-like protease, leading to abnormal desquamation. On the other hand, an elevated pH favors other proteases, such as kallikrein-related peptidases 5 and 7, which are both expressed in the epidermis [104–107]. Elevation in skin surface pH can activate kallikrein-related peptidase 5, leading to the development of atopic dermatitis-like lesions in mice via proteinase-activated receptor-2 dependent and independent pathways [108–110]. Moreover, kallikrein-related peptidase 7 can activate IL-1β [reviewed in 111]. Overexpression of epidermal kallikrein-related peptidase 7 results in the development of cutaneous inflammation [112]. Taken together, the elevated skin surface pH can contribute to the development of inflammation, infections and compromised permeability barrier homeostasis in aged skin.

## Approaches to emprove epidermal function in aged skin

Because of the substantial impact of epidermal dysfunctions on cutaneous and extracutaneous function, great efforts have been made to develop regimens to improve epidermal functions in chronologically-aged skin. Several approaches have been proven to benefit epidermal functions in aged mice and/or humans.

### Acidification of the stratum corneum

Studies have demonstrated that acidification of the stratum corneum alone can improve epidermal structure and permeability barrier homeostasis in aged skin. For example, acidification of aged mouse skin with topical lactobionic acid normalized permeability barrier homeostasis and the structure of corneodesmosomes in the stratum corneum [36]. Likewise, topical applications of an emollient at pH 4.0 for 29 days markedly improve stratum corneum hydration and lamellar bilayer structure, along with increased resistance to challenges from topical sodium dodecyl sulphate, in aged humans [113]. Similarly, compared to a pH 5.8 emollient, a topical pH 4.0 emollient accelerates permeability barrier recovery following acute disruption, and significantly improves stratum corneum integrity after 28-day treatments on aged humans [114]. Although acidifying the stratum corneum could prevent and alleviate atopic dermatitis-like skin lesions in young mice [115–118], whether the same benefits could be achieved in aged humans remains to be determined. Nonetheless, acidification of the stratum corneum can improve epidermal structure and function in chronologically-aged humans.

### Topical applications of stratum corneum lipids

Reductions in stratum corneum lipid content can largely contribute to the delayed permeability barrier recovery in aged skin [7, 14]. Accordingly, topical applications of a lipid mixture containing three key stratum corneum lipids; i.e., cholesterol, free fatty acids and ceramides, accelerate permeability barrier recovery in both aged mice and humans [27]. A recent study demonstrated that topical applications of an emollient containing stratum corneum lipids not only improved epidermal permeability barrier, stratum corneum hydration and skin surface pH, but also lowered circulating levels of proinflammatory cytokines in aged humans [5]. There are at least two possible mechanisms by which the topical lipid mixture improves epidermal function. One is that topical lipids penetrate into keratinocytes in the stratum granulosum, where they are packaged in lamellar bodies, then secreted into the stratum corneum, where lamellar bilayers are formed [23, 119]. The other mechanism is that this lipid mixture contains fatty acids, which can activate peroxisome proliferator-activated receptors (PPAR) [120]. Activation of PPARs stimulates epidermal lipid production and keratinocyte differentiation, resulting in improvement in epidermal permeability barrier function and inhibition of cutaneous inflammation [121–123]. Thus, topical applications of stratum corneum lipid mixtures not only provide lipids for membrane bilayer formation, but also upregulate keratinocyte function, leading to improvements in epidermal function. However, whether topical PPAR ligands alone can improve epidermal functions in the elderly has not been sufficiently proved. Haratake et al. [124] showed that topical application of either cholesterol or mevalonic acid, a precursor of cholesterol, improved the stratum corneum integrity and epidermal permeability barrier recovery in mice. Whether topical cholesterol or mevalonic acid alone can improve epidermal permeability barrier in the aged humans remains to be determined.

### Natural ingredients

Studies have shown that several natural ingredients can improve epidermal function in aged humans and mice. For example, oral administration of either vitamin C or linoleic acid improved senile xerosis [125]. Likewise, orally-taken wheat extract oil increased stratum corneum hydration [126, 127], while orally-taken milk, containing green tea extract, borage oil and vitamin E, improved epidermal permeability barrier [128]. Similarly, oral supplements of flaxseed and borage oil increased stratum corneum hydration, while decreasing transepidermal water loss rates in humans [129]. In addition, estrogen replacement can improve multiple epidermal functions, including permeability barrier homeostasis, stratum corneum hydration and stratum corneum integrity, in both mice and humans [130, 131]. Besides oral administration, topical applications of some natural ingredients can also improve epidermal functions in chronologically-aged skin. Man et al. [79] reported that topical applications of hesperidin improved epidermal functions, including reductions in skin surface pH, acceleration of permeability barrier recovery and stimulation of keratinocyte differentiation, in aged mice. Other natural ingredients, such as petrolatum, glycerol, coconut oil and sunflower oil, can also improve stratum corneum hydration and epidermal permeability barrier [132–136]. Interestingly, bathing with soybean oil could also decrease transepidermal water loss, while increasing stratum corneum hydration, in comparison to bathing without soybean oil [137]. In addition to improving epidermal function, topical petrolatum and glycerol could lower circulating levels of cytokines in aged mice, too [87]. Taken together, either oral or topical administration of certain natural ingredients can improve epidermal function in aged skin.

It appears that the influence of some natural ingredients on epidermal functions is due to upregulation of keratinocyte function. For instance, topical hesperidin can upregulate expression levels of mRNA related to epidermal differentiation, lipid production and acidification in aged skin [79]. Likewise, topical applications of a mixture of several plant oils increase ceramide content, along with improvements in stratum corneum hydration and epidermal permeability barrier function in aged skin [138]. Similarly, topical applications of bacterial sphingomyelinase from *Streptococcus thermophiles* also increase stratum corneum hydration and stratum corneum ceramide content in aged humans [139]. Moreover, glycerol and petrolatum, which are traditionally viewed as inert ingredients, can also stimulate keratinocyte differentiation [140, 141]. Of course, water holding and occlusive properties of glycerol and petrolatum also contribute to enhanced epidermal permeability barrier and stratum corneum hydration.

In summary, chronologically-aged skin displays multiple alterations in epidermal functions, which can contribute to the development of a number of cutaneous and extracutaneous disorders. Accordingly, improvements in epidermal function can be a valuable alternative to prevent and ameliorate disorders, which are linked to epidermal dysfunction in the elderly. A wide range of ingredients can improve epidermal function. However, it is worth noting that a substantial portion of emollients on the market are harmful to epidermal function, although some ingredients in these products may benefit epidermal function [142–145]. The harmful emollients often contain some ingredient, such as eicosadienoic acid-enriched oils, stearic acid, ceteareth 20, PEG-40 castor oil and PEG-100 stearate, which all can induce cutaneous inflammation and/or disrupt epidermal permeability barrier [145–148]. Use of harmful emollients, especially in the long-term, could compromise epidermal function, leading to the development and exacerbation of some cutaneous and extracutaneous disorders associated with epidermal dysfunction. Therefore, caution should be taken when choosing emollients.
